# Convergence Analysis for a Modified SP Iterative Method

**DOI:** 10.1155/2014/840504

**Published:** 2014-08-10

**Authors:** Fatma Öztürk Çeliker

**Affiliations:** Department of Mathematics, Yildiz Technical University, Davutpasa Campus, Esenler, 34220 Istanbul, Turkey

## Abstract

We consider a new iterative method due to Kadioglu and Yildirim (2014) for further investigation. We study convergence analysis of this iterative method when applied to class of contraction mappings. Furthermore, we give a data dependence result for fixed point of contraction mappings with the help of the new iteration method.

## 1. Introduction

Recent progress in nonlinear science reveals that iterative methods are most powerful tools which are used to approximate solutions of nonlinear problems whose solutions are inaccessible analytically. Therefore, in recent years, an intensive interest has been devoted to developing faster and more effective iterative methods for solving nonlinear problems arising from diverse branches in science and engineering.

Very recently the following iterative methods are introduced in [1] and [2], respectively:

(1) $$\begin{array}{c} x_{0}\in D,\\ x_{n+1}=Ty_{n},\\ y_{n}=\left(1-\alpha_{n}\right)z_{n}+\alpha_{n}Tz_{n},\\ z_{n}=\left(1-\beta_{n}\right)x_{n}+\beta_{n}Tx_{n},\;n\in N, \end{array}$$

(2) $$\begin{array}{c} u_{0}\in D,\\ u_{n+1}=Tv_{n},\\ v_{n}=\left(1-\alpha_{n}\right)Tu_{n}+\alpha_{n}Tw_{n},\\ w_{n}=\left(1-\beta_{n}\right)u_{n}+\beta_{n}Tu_{n},\;n\in N, \end{array}$$

where *D* is a nonempty convex subset of a Banach space *B*, *T* is a self map of *D*, and {*α*_*n*_}_*n*=0_^*∞*^, {*β*_*n*_}_*n*=0_^*∞*^ are real sequences in [0,1].

While the iterative method (1) fails to be named in [1], the iterative method (2) is called Picard-S iteration method in [2]. Since iterative method (1) is a special case of SP iterative method of Phuengrattana and Suantai [3], we will call it here Modified SP iterative method.

It was shown in [1] that Modified SP iterative method (1) is faster than all Picard [4], Mann [5], Ishikawa [6], and *S* [7] iterative methods in the sense of Definitions 1 and 2 given below for the class of contraction mappings satisfying

(3) $$\begin{array}{c} ||Tx-Ty||\leq \delta ||x-y||,\;\delta \in \left(0,1\right),\;\forall x,y\in B. \end{array}$$

Using the same class of contraction mappings (3), Gürsoy and Karakaya [2] showed that Picard-S iteration method (2) is also faster than all Picard [4], Mann [5], Ishikawa [6], *S* [7], and some other iterative methods in the existing literature.

In this paper, we show that Modified SP iterative method converges to the fixed point of contraction mappings (3). Also, we establish an equivalence between convergence of iterative methods (1) and (2). For the sake of completness, we give a comparison result between the rate of convergences of iterative methods (1) and (2), and it thus will be shown that Picard-S iteration method is still the fastest method. Finally, a data dependence result for the fixed point of the contraction mappings (3) is proven.

The following definitions and lemmas will be needed in order to obtain the main results of this paper.

**Definition 1 (see [8]).**

Let {*a*_*n*_}_*n*=0_^*∞*^ and {*b*_*n*_}_*n*=0_^*∞*^ be two sequences of real numbers with limits *a* and *b*, respectively. Suppose that

(4) $$\begin{array}{c} \underset{n\to \infty }{\lim}\frac{\left|a_{n}-a\right|}{\left|b_{n}-b\right|}=l \end{array}$$

exists.

- If *l* = 0, then we say that {*a*_*n*_}_*n*=0_^*∞*^ converges faster to *a* than {*b*_*n*_}_*n*=0_^*∞*^ to *b*.
- If 0 < *l* < *∞*, then we say that {*a*_*n*_}_*n*=0_^*∞*^ and {*b*_*n*_}_*n*=0_^*∞*^ have the same rate of convergence.

**Definition 2 (see [8]).**

Assume that for two fixed point iteration processes {*u*_*n*_}_*n*=0_^*∞*^ and {*v*_*n*_}_*n*=0_^*∞*^ both converging to the same fixed point *p*, the following error estimates,

(5) $$\begin{array}{c} ||u_{n}-p||\leq a_{n}\;\forall n\in N,\\ ||v_{n}-p||\leq b_{n}\;\forall n\in N, \end{array}$$

are available where {*a*_*n*_}_*n*=0_^*∞*^ and {*b*_*n*_}_*n*=0_^*∞*^ are two sequences of positive numbers (converging to zero). If {*a*_*n*_}_*n*=0_^*∞*^ converges faster than {*b*_*n*_}_*n*=0_^*∞*^, then {*u*_*n*_}_*n*=0_^*∞*^ converges faster than {*v*_*n*_}_*n*=0_^*∞*^ to *p*.

**Definition 3 (see [9]).**

Let $T,\tilde{T}:B\to B$ be two operators. We say that $\tilde{T}$ is an approximate operator of *T* if for all *x* ∈ *B* and for a fixed *ε* > 0 we have

(6) $$\begin{array}{c} ||Tx-\tilde{T}x||\leq \varepsilon . \end{array}$$

**Lemma 4 (see [10]).**

Let {*σ*_*n*_}_*n*=0_^*∞*^ and {*ρ*_*n*_}_*n*=0_^*∞*^ be nonnegative real sequences and suppose that for all *n* ≥ *n*_0_, *τ*_*n*_ ∈ (0,1), ∑_*n*=1_^*∞*^ *τ*_*n*_ = *∞*, and *ρ*_*n*_/*τ*_*n*_ → 0 as *n* → *∞*

(7) $$\begin{array}{c} \sigma_{n+1}\leq \left(1-\tau_{n}\right)\sigma_{n}+\rho_{n} \end{array}$$

holds. Then lim⁡_*n*→*∞*_ *σ*_*n*_ = 0.

**Lemma 5 (see [11]).**

Let {*σ*_*n*_}_*n*=0_^*∞*^ be a nonnegative sequence such that there exists *n*_0_ ∈ *N*, for all *n* ≥ *n*_0_; the following inequality holds. Consider

(8) $$\begin{array}{c} \sigma_{n+1}\leq \left(1-\tau_{n}\right)\sigma_{n}+\tau_{n}\mu_{n}, \end{array}$$

where *τ*_*n*_ ∈ (0,1), for all *n* ∈ *N*, ∑_*n*=0_^*∞*^ *τ*_*n*_ = *∞* and *η*_*n*_ ≥ 0, ∀*n* ∈ *N*. Then

(9) $$\begin{array}{c} 0\leq \underset{}{\lim}\underset{n\to \infty }{\sup}\;\sigma_{n}\leq \underset{}{\lim}\underset{n\to \infty }{\sup}\;\mu_{n}. \end{array}$$

## 2. Main Results

**Theorem 6 .**

Let *D* be a nonempty closed convex subset of a Banach space *B* and *T* : *D* → *D* a contraction map satisfying condition (3). Let {*x*_*n*_}_*n*=0_^*∞*^ be an iterative sequence generated by (1) with real sequences {*α*_*n*_}_*n*=0_^*∞*^, {*β*_*n*_}_*n*=0_^*∞*^ in [0,1] satisfying ∑_*k*=0_^*∞*^ *α*_*k*_ = *∞*. Then {*x*_*n*_}_*n*=0_^*∞*^ converges to a unique fixed point of *T*, say *x*_∗_.

**Proof**

The well-known Picard-Banach theorem guarantees the existence and uniqueness of *x*_∗_. We will show that *x*_*n*_ → *x*_∗_ as *n* → *∞*. From (3) and (1) we have

(10) ||xn+1−x∗||=||Tyn−Tx∗||≤δ||yn−x∗||≤δ{(1−αn)||zn−x∗||+αnδ||zn−x∗||}≤δ[1−αn(1−δ)]||zn−x∗||≤δ[1−αn(1−δ)]×{(1−βn)||xn−x∗||+βnδ||xn−x∗||}≤δ[1−αn(1−δ)][1−βn(1−δ)]||xn−x∗||≤δ[1−αn(1−δ)]||xn−x∗||.

By induction on the inequality (10), we derive

(11) ||xn+1−x∗||≤||x0−x∗||δn+1∏k=0n[1−αk(1−δ)]≤||x0−x∗||δn+1e−(1−δ)∑k=0nαk.

Since ∑_*k*=0_^*∞*^ *α*_*k*_ = *∞*, taking the limit of both sides of inequality (11) yields lim⁡_*n*→*∞*_||*x*_*n*_ − *x*_∗_|| = 0; that is, *x*_*n*_ → *x*_∗_ as *n* → *∞*.

**Theorem 7 .**

Let *D*, *B*, and *T* with fixed point *x*_∗_ be as in Theorem 6. Let {*x*_*n*_}_*n*=0_^*∞*^, {*u*_*n*_}_*n*=0_^*∞*^ be two iterative sequences defined by (1) and (2), respectively, with real sequences {*α*_*n*_}_*n*=0_^*∞*^, {*β*_*n*_}_*n*=0_^*∞*^ in [0,1] satisfying ∑_*k*=0_^*∞*^ *α*_*k*_ *β*_*k*_ = *∞*. Then the following are equivalent:

- {*x*_*n*_}_*n*=0_^*∞*^ converges to *x*_∗_;
- {*u*_*n*_}_*n*=0_^*∞*^ converges to *x*_∗_.

**Proof**

We will prove (i)⇒(ii). Now by using (1), (2), and condition (3), we have

(12) ||xn+1−un+1||=||Tyn−Tvn||≤δ||yn−vn||=δ||(1−αn)zn+αnTzn−(1−αn)Tun−αnTwn||≤δ{(1−αn)||zn−Tzn||+(1−αn)δ||zn−un||+αnδ||zn−wn||}≤(1−αn)δ||xn−un||+(1−αn)δβn||Txn−xn||+αnδ||zn−wn||+(1−αn)||zn−Tzn||≤(1−αn)δ||xn−un||+(1−αn)δβn||Txn−xn||+αnδ[1−βn(1−δ)]||xn−un||+(1−αn)||zn−Tzn||≤[1−αn(1−δ)]||xn−un||+(1−αn)δβn||Txn−xn||+(1−αn)||zn−Tzn||.

Define

(13) σn:=||xn−un||,τn:=αn(1−δ)∈(0,1),ρn:=(1−αn)δβn||xn−Txn||+(1−αn)||zn−Tzn||.

Since lim⁡_*n*→*∞*_||*x*_*n*_ − *x*_∗_|| = 0 and *Tx*_∗_ = *x*_∗_, lim⁡_*n*→*∞*_||*x*_*n*_ − *Tx*_*n*_|| = lim⁡_*n*→*∞*_||*z*_*n*_ − *Tz*_*n*_|| = 0 which implies *ρ*_*n*_/*τ*_*n*_ → 0 as *n* → *∞*. Since also *α*_*n*_, *β*_*n*_ ∈ [0,1] for all *n* ∈ *N*

(14) $$\begin{array}{c} \alpha_{n}\beta_{n}<\alpha_{n}; \end{array}$$

hence the assumption ∑_*k*=0_^*∞*^ *α*_*k*_ *β*_*k*_ = *∞* leads to

(15) $$\begin{array}{c} \overset{\infty }{\underset{k=0}{\sum }}\alpha_{k}=\infty . \end{array}$$

Thus all conditions of Lemma 4 are fulfilled by (12), and so lim⁡_*n*→*∞*_||*x*_*n*_ − *u*_*n*_|| = 0. Since

(16) $$\begin{array}{c} ||u_{n}-x_{*}||\leq ||x_{n}-u_{n}||+||x_{n}-x_{*}||,\\ \underset{n\to \infty }{\lim}||u_{n}-x_{*}||=0. \end{array}$$

Using the same argument as above one can easily show the implication (ii)⇒(i); thus it is omitted here.

**Theorem 8 .**

Let *D*, *B*, and *T* with fixed point *x*_∗_ be as in Theorem 6. Let {*α*_*n*_}_*n*=0_^*∞*^, {*β*_*n*_}_*n*=0_^*∞*^ be real sequences in (0,1) satisfying

- lim⁡_*n*→*∞*_ *α*_*n*_ = lim⁡_*n*→*∞*_ *β*_*n*_ = 0.

For given *x*_0_ = *u*_0_ ∈ *D*, consider iterative sequences {*x*_*n*_}_*n*=0_^*∞*^ and {*u*_*n*_}_*n*=0_^*∞*^ defined by (1) and (2), respectively. Then {*u*_*n*_}_*n*=0_^*∞*^ converges to *x*_∗_ faster than {*x*_*n*_}_*n*=0_^*∞*^ does.

**Proof**

The following inequality comes from inequality (10) of Theorem 6:

(17) ||xn+1−x∗||≤||x0−x∗||δn+1×∏k=0n[1−αk(1−δ)][1−βk(1−δ)].

The following inequality is due to ([2], inequality (2.5) of Theorem 1):

(18) ||un+1−x∗||≤||u0−x∗||δ2(n+1)×∏k=0n[1−αkβk(1−δ)].

Define

(19) an:=||u0−x∗||δ2(n+1)∏k=0n[1−αkβk(1−δ)],bn:=||x0−x∗||δn+1∏k=0n[1−αk(1−δ)][1−βk(1−δ)].

Since *x*_0_ = *u*_0_

(20) $$\begin{array}{c} \theta_{n}:=\frac{a_{n}}{b_{n}}=\frac{\delta^{n+1}\prod_{k=0}^{n}\left[1-\alpha_{k}\beta_{k}\left(1-\delta \right)\right]}{\prod_{k=0}^{n}\left[1-\alpha_{k}\left(1-\delta \right)\right]\left[1-\beta_{k}\left(1-\delta \right)\right]}. \end{array}$$

Therefore, taking into account assumption (i), we obtain

(21) lim⁡n→∞θn+1θn=lim⁡n→∞δ[1−αn+1βn+1(1−δ)][1−αn+1(1−δ)][1−βn+1(1−δ)]=δ<1.

It thus follows from well-known ratio test that ∑_*n*=0_^*∞*^ *θ*_*n*_ < *∞*. Hence, we have lim⁡_*n*→*∞*_ *θ*_*n*_ = 0 which implies that {*u*_*n*_}_*n*=0_^*∞*^ is faster than {*x*_*n*_}_*n*=0_^*∞*^.

In order to support analytical proof of Theorem 8 and to illustrate the efficiency of Picard-S iteration method (2), we will use a numerical example provided by Sahu [12] for the sake of consistent comparison.

**Example 9 .**

Let *B* = *R* and *D* = [0, *∞*). Let *T* : *D* → *D* be a mapping and for all $x\in D,Tx=\sqrt[{3}]{3x+18}$ . *T* is a contraction with contractivity factor $\delta =1/\sqrt[{3}]{18}$ and *x*_∗_ = 3; see [12]. Take *α*_*n*_ = *β*_*n*_ = *γ*_*n*_ = 1/(*n* + 1) with initial value *x*_0_ = 1000. Tables 1, 2, and 3 show that Picard-S iteration method (2) converges faster than all SP [3], Picard [4], Mann [5], Ishikawa [6], *S* [7], CR [13], S* [14], Noor [15], and Normal-*S* [16] iteration methods including a new three-step iteration method due to Abbas and Nazir [17].

We are now able to establish the following data dependence result.

**Theorem 10 .**

Let $\tilde{T}$ be an approximate operator of *T* satisfying condition (3). Let {*x*_*n*_}_*n*=0_^*∞*^ be an iterative sequence generated by (1) for *T* and define an iterative sequence ${\left\{{\tilde{x}}_{n}\right\}}_{n=0}^{\infty }$ as follows:

(22) $$\begin{array}{c} {\tilde{x}}_{0}\in D,\\ {\tilde{x}}_{n+1}=\tilde{T}{\tilde{y}}_{n},\\ {\tilde{y}}_{n}=\left(1-\alpha_{n}\right){\tilde{z}}_{n}+\alpha_{n}\tilde{T}{\tilde{z}}_{n},\\ {\tilde{z}}_{n}=\left(1-\beta_{n}\right){\tilde{x}}_{n}+\beta_{n}\tilde{T}{\tilde{x}}_{n},\;n\in N, \end{array}$$

where {*α*_*n*_}_*n*=0_^*∞*^, {*β*_*n*_}_*n*=0_^*∞*^ are real sequences in [0,1] satisfying (i) 1/2 ≤ *α*_*n*_, (ii) *β*_*n*_ ≤ *α*_*n*_ for all *n* ∈ *N*, and (iii) ∑_*n*=0_^*∞*^ *α*_*n*_ = *∞*. If *Tx*_∗_ = *x*_∗_ and $\tilde{T}{\tilde{x}}_{*}={\tilde{x}}_{*}$ such that ${\tilde{x}}_{n}\to {\tilde{x}}_{*}$ as *n* → *∞*, then we have

(23) $$\begin{array}{c} ||x_{*}-{\tilde{x}}_{*}||\leq \frac{4\varepsilon }{1-\delta }, \end{array}$$

where *ε* > 0 is a fixed number and *δ* ∈ (0,1).

**Proof**

It follows from (1), (3), (22), and assumption (ii) that

(24) ||xn+1−x~n+1||=||Tyn−Ty~n+Ty~n−T~y~n||≤||Tyn−Ty~n||+||Ty~n−T~y~n||≤δ||yn−y~n||+ε≤δ(1−αn)||zn−z~n||+δαn||Tzn−Tz~n||+δαn||Tz~n−T~z~n||+ε≤δ[1−αn(1−δ)]||zn−z~n||+δαnε+ε≤δ[1−αn(1−δ)][1−βn(1−δ)]||xn−x~n||+δ[1−αn(1−δ)]βnε+δαnε+ε≤[1−αn(1−δ)]||xn−x~n||+2αnε+ε.

From assumption (i) we have

(25) $$\begin{array}{c} 1\leq 2\alpha_{n}, \end{array}$$

and thus, inequality (24) becomes

(26) ||xn+1−x~n+1||≤[1−αn(1−δ)]||xn−x~n||+4αnε≤[1−αn(1−δ)]||xn−x~n||+αn(1−δ)4ε1−δ.

Denote that

(27) $$\begin{array}{c} \sigma_{n}:=||x_{n}-{\tilde{x}}_{n}||,\;\;\tau_{n}:=\alpha_{n}\left(1-\delta \right)\in \left(0,1\right),\\ \mu_{n}:=\frac{4\varepsilon }{1-\delta }. \end{array}$$

It follows from Lemma 5 that

(28) $$\begin{array}{c} 0\leq \underset{n\to \infty }{\lim\;\sup}||x_{n}-{\tilde{x}}_{n}||\leq \underset{n\to \infty }{\lim\;\sup}\;\frac{4\varepsilon }{1-\delta }. \end{array}$$

From Theorem 6 we know that lim⁡_*n*→*∞*_ *x*_*n*_ = *x*_∗_. Thus, using this fact together with the assumption $\lim_{n\to \infty }{\tilde{x}}_{n}={\tilde{x}}_{*}$ we obtain

(29) $$\begin{array}{c} ||x_{*}-{\tilde{x}}_{*}||\leq \frac{4\varepsilon }{1-\delta }. \end{array}$$

## Figures and Tables

**Table 1:** Comparison speed of convergence among various iteration methods.

Number of iterations	Picard-S	Abbas and Nazir	Modified SP	*S* ∗
1	3.101431265	3.944094141	3.101431265	3.101431265
2	3.000970459	3.032885422	3.003472396	3.006099262
3	3.000010797	3.001099931	3.000191044	3.000474311
4	3.000000126	3.000033381	3.000012841	3.000040908
5	3.000000001	3.000000928	3.000000964	3.000003733
6	3.000000000	3.000000024	3.000000078	3.000000354
7	3.000000000	3.000000000	3.000000007	3.000000034
8	3.000000000	3.000000000	3.000000001	3.000000004
9	3.000000000	3.000000000	3.000000000	3.000000000
⋮	⋮	⋮	⋮	⋮

**Table 2:** Comparison speed of convergence among various iteration methods.

Number of iterations	CR	Normal *S*	*S*	Picard
1	3.101431265	3.944094141	3.944094141	14.45128320
2	3.004853706	3.056995075	3.079213170	3.944094141
3	3.000341967	3.004449310	3.007910488	3.101431265
4	3.000027911	3.000384457	3.000829879	3.011228065
5	3.000002459	3.000035123	3.000088928	3.001247045
6	3.000000227	3.000003324	3.000009637	3.000138554
7	3.000000022	3.000000323	3.000001051	3.000015395
8	3.000000003	3.000000032	3.000000115	3.000001710
9	3.000000000	3.000000003	3.000000013	3.000000190
10	3.000000000	3.000000000	3.000000001	3.000000021
11	3.000000000	3.000000000	3.000000000	3.000000002
12	3.000000000	3.000000000	3.000000000	3.000000000
⋮	⋮	⋮	⋮	⋮

**Table 3:** Comparison speed of convergence among various iteration methods.

Number of	SP	Noor	Ishikawa	Mann
iterations				
1	3.101431265	3.101431265	3.944094141	14.45128320
2	3.017380074	3.053700718	3.500544608	9.197688670
3	3.006056041	3.037176288	3.346563527	7.322609407
4	3.002849358	3.028678163	3.267333303	6.346715746
5	3.001583841	3.023463545	3.218710750	5.744057924
6	3.000979045	3.019921623	3.185684999	5.333095485
7	3.000651430	3.017350921	3.161716071	5.034023149
8	3.000457519	3.015395770	3.143487338	4.806124994
9	3.000334906	3.013856108	3.129133091	4.626397579
10	3.000253300	3.012610550	3.117521325	4.480838008
11	3.000196722	3.011581068	3.107924338	4.360421594
12	3.000156165	3.010715155	3.099852480	4.259063398
13	3.000126272	3.009976148	3.092963854	4.172507477
⋮	⋮	⋮	⋮	⋮

## References

[1] Kadioglu N, Yildirim I Approximating fixed points of nonexpansive mappings by a faster iteration process. http://arxiv.org/abs/1402.6530.

[2] Gürsoy F, Karakaya V A Picard-S hybrid type iteration method for solving a differential equation with retarded argument. http://arxiv.org/abs/1403.2546.

[3] Phuengrattana W, Suantai S (2011). On the rate of convergence of Mann, Ishikawa, Noor and SP-iterations for continuous functions on an arbitrary interval. *Journal of Computational and Applied Mathematics*.

[4] Picard E (1890). Mémoire sur la théorie des équations aux dérivées partielles et la méthode des approximations successives. *Journal de Mathématiques pures et appliquées*.

[5] Mann WR (1953). Mean value methods in iteration. *Proceedings of the American Mathematical Society*.

[6] Ishikawa S (1974). Fixed points by a new iteration method. *Proceedings of the American Mathematical Society*.

[7] Agarwal R, O Regan D, Sahu D (2007). Iterative construction of fixed points of nearly asymptotically nonexpansive mappings. *Journal of Nonlinear and Convex Analysis*.

[8] Berinde V (2004). Picard iteration converges faster than Mann iteration for a class of quasi-contractive operators. *Fixed Point Theory and Applications*.

[9] Berinde V (2007). *Iterative Approximation of Fixed Points*.

[10] Weng X (1991). Fixed point iteration for local strictly pseudo-contractive mapping. *Proceedings of the American Mathematical Society*.

[11] Şoltuz ŞM, Grosan T (2008). Data dependence for Ishikawa iteration when dealing with contractive-like operators. *Fixed Point Theory and Applications*.

[12] Sahu DR (2011). Applications of the S-iteration process to constrained minimization problems and split feasibility problems. *Fixed Point Theory*.

[13] Chugh R, Kumar V, Kumar S (2012). Strong convergence of a new three step iterative scheme in Banach spaces. *The American Journal of Computational Mathematics*.

[14] Karahan I, Ozdemir M (2013). A general iterative method for approximation of fixed points and their applications. *Advances in Fixed Point Theory*.

[15] Noor MA (2000). New approximation schemes for general variational inequalities. *Journal of Mathematical Analysis and Applications*.

[16] Sahu DR, Petruşel A (2011). Strong convergence of iterative methods by strictly pseudocontractive mappings in Banach spaces. *Nonlinear Analysis: Theory, Methods & Applications*.

[17] Abbas M, Nazir T (2014). A new faster iteration process applied to constrained minimization and feasibility problems. *Matematicki Vesnik*.

